# Noninfectious Causes of Fever in 128 Patients with Systemic Lupus Erythematosus

**Published:** 2019-01

**Authors:** Feng GUO, Jianmei CHEN, Yu XIE, Xueping ZHOU

**Affiliations:** 1. Department of Rheumatism, Affiliated Hospital of Nanjing University of Traditional Chinese Medicine, Nanjing 210029, China; 2. Nanjing University of Chinese Medicine, Nanjing 210023, P.R. China

**Keywords:** Lupus erythematosus, Systemic, Fever, Risk factors, Retrospective study

## Abstract

**Background:**

This study aimed to explore the clinical characteristics of noninfectious fever in patients with systemic lupus erythematosus (SLE) and the underlying causes through multivariate analysis.

**Methods:**

Clinical data of 128 patients with SLE who were admitted to Affiliated Hospital of Nanjing University of TCM, Nanjing 210029, P.R. China from January 2015 to December 2017 were retrospectively analyzed.

**Results:**

The following factors were closely associated with fever: patient age, treatment history, SLEDAI score, enlarged liver, spleen and lymph nodes, low hemoglobin, leukopenia, CRP, complement C3, albumin, anti-dsDNA antibody, glucocorticoids, and cyclophosphamide. Multivariate logistic regression analysis showed that factors, such as enlarged liver, spleen and lymph nodes, digestive system involvement, low hemoglobin, leukopenia, CRP, decreased albumin, anti-dsDNA antibody, glucocorticoids and cyclophosphamide, were closely associated with noninfectious fever in SLE.

**Conclusion:**

Noninfectious fever is a common clinical manifestation of SLE. Factors, such as enlarged liver, spleen and lymph nodes, digestive system involvement, low hemoglobin, leukopenia, CRP, decreased albumin, anti-dsDNA antibody, glucocorticoids, and cyclophosphamide, were independent risk factors for noninfectious fever in SLE.

## Introduction

Systemic lupus erythematosus (SLE) is an autoimmune disease with high morbidity and mortality that involves multiple systems and multiple organs (1). There are several immunological abnormalities typical of SLE, including defects of phagocytic function, decreased chemotaxis of leukocytes, complement deficiency, decreased lymphocyte cytokines, and macrophage and NK cell dysfunction (2).

The clinical manifestations of SLE are diverse, with common systemic symptoms ranging from moderate to severe fatigue, weight loss and fever. The fever pattern in SLE is irregular, and a low-grade fever is more common than others. When the condition deteriorates, a high fever is often seen and frequently accompanied with chills and headache. Clinically, it is often necessary to make a differential diagnosis of the fever in SLE to determine whether it is caused by infection or disease activity (3, 4). This is especially true for patients with SLE who are long-term users of glucocorticoids and immunosuppressive agents, because these patients often have fever episodes caused by infection. Early monitoring and assessment of fever are critically important since the medications used in fever treatment are completely different depending on the underlying causes.

In this study, the clinical data of 128 patients with noninfectious SLE who were admitted to Affiliated Hospital of Nanjing University of TCM were retrospectively analyzed. The clinical characteristics of noninfectious fever in SLE were explored by multivariate analysis of various relevant factors. The goal of this study was to provide guidance on differential diagnosis, prevention and treatment of noninfectious fever in SLE.

## Subjects and methods

### Subjects

A total of 128 patients with SLE admitted to Affiliated Hospital of Nanjing University of TCM, Nanjing, P.R. China from January 2015 to December 2017 were followed up. Relevant clinical data were collected. There were 8 males and 120 females aged 14–62 yr with an average age of 34.9±11 years. The patients were divided into two groups: 53 in noninfectious fever group and 75 in no-fever group. In the noninfectious fever group, there were 4 males and 49 females with an average age of 32.5±9.4 years. In the no-fever group, there were 4 males and 71 females with an average age of 36.7±11.8 years. The patient inclusion criteria were in accordance with the 1997 update of the 1982 American College of Rheumatology revised criteria for classification of systemic lupus erythematosus. Patients who had the following conditions were excluded from the study: infection, tumors, post-operative stress fever, transfusion reactions or infusion reactions. In this study, a fever was defined as an oral temperature above 37.5°C.

The collection of all clinical data has been approved by the patients and their family and signed informed consent. This study was approved by the Ethics Committee of Affiliated Hospital of Nanjing University of TCM.

### Methods

Relevant clinical data were obtained through retrospective collection of existing medical record information, supplemented by outpatient review and telephone follow-up calls. The following clinical manifestations were compared in this study: photosensitivity, arthritis, serositis, enlarged liver, spleen and lymph nodes, digestive system involvement, skin and mucosal involvement, and neuropsychiatric involvement. Digestive system involvement referred to at least one condition of dyspepsia, intestinal pseudo-obstruction, mesenteric vasculitis, protein-losing enteropathy, and pancreatitis. Neuropsychiatric involvement referred to at least one condition of encephalitis, cerebrovascular accident, cranial neuropathy, peripheral neuropathy, transverse myelitis, epilepsy, and psychosis. Abnormal laboratory test results were defined as: hemoglobin of less than 100 g/L, platelet count of less than 80 × 10^9^/L, white blood cell count of less than 4.0 × 10^9^ /L, C-reactive protein (CRP) of greater than 8 mg/L, alanine transaminase (ALT) of greater than 60 U/L, serum creatinine of greater than 130 μmol/L, and albumin of less than 35 g/L. The following common drugs used in clinical treatment of SLE were given in this study: glucocorticoids, cyclophosphamide, hydroxychloroquine/chloroquine, azathioprine/methotrexate, and tripterygium glycosides. The clinical manifestations, laboratory test results, and received medications were compared between patients in the two groups.

### Statistical analysis

The statistics software SPSS 22.0 (Chicago, IL, USA) was used in the statistical analysis. The measurement data were expressed as mean ± standard deviation and analyzed using the *t* test. The count data were analyzed using the χ^2^ test. Correlation analysis was performed using the multivariate logistic regression model. The difference was statistically significant when *P*<0.05.

## Results

### General clinical data of patients in the noninfectious fever group and the no-fever group

A total of 128 patients with SLE met the inclusion criteria and were divided into two groups: 53 in noninfectious fever group (abbreviated as fever group) and 75 in no-fever group. A statistical analysis was performed on the following items of the general clinical data: age, gender, family history, initial treatment, number of hospitalizations, and SLE disease activity index (SLEDAI) score. As shown in Table 1, the differences in age (*P*=0.035), initial treatment (*P*=0.045), and the SLEDAI score (*P*=0.008) were statistically significant between patients in the two groups, while the differences in gender (*P*=0.317), family history (*P*=0.569), and number of hospitalizations (*P*=0.282) were not statistically significant.

**Table 1:** General clinical data of patients in the fever group and the no-fever group

***General data***	***Fever group (n=53)***	***No-fever group (n=75)***	***X ^ 2 ^ /T Value***	***P Value***
Age (yr)	32.5±9.4	36.7±11.8	−2.132	0.035
Gender (female)	49 (92.5%)	71 (94.7%)	1.546	0.317
Family history (yes)	2 (3.8%)	1 (1.3%)	0.808	0.569
Initial treatment	23 (43.4%)	46 (61.3%)	4.021	0.045
Number of hospitalizations	1.7±0.8	1.6±0.9	1.080	0.282
SLEDAI score	13.3±4.1	11.3±4.1	2.708	0.008

### Clinical manifestations of SLE in the noninfectious fever group and the no-fever group

The clinical manifestations of SLE in the noninfectious fever group and the no-fever group were compared. The difference in enlarged liver, spleen and lymph nodes was statistically significant between the two groups (*P*=0.036), while the differences in photosensitivity (*P*=0.604), arthritis (*P*=0.779), serositis (*P*=0.282), digestive system involvement (*P*=0.714), skin and mucosal involvement (*P*=0.097), and neuropsychiatric involvement (*P*=0.111) were not statistically significant (*P*>0.05) (Table 2).

**Table 2:** Clinical manifestations of SLE in the noninfectious fever group and the no-fever group

***Clinical manifestations***	***Fever group (n=53) %***	***No-fever group (n=75) %***	***X ^ 2 ^ /T Value***	***P Value***
Photosensitivity	10 (18.9)	17 (22.7)	0.269	0.604
Arthritis	31 (58.4)	42 (56.0)	0.079	0.779
Serositis	10 (18.9)	9 (12.0)	1.159	0.282
Enlarged liver, spleen and lymph nodes	11 (20.8)	6 (8.0)	4.386	0.036
Digestive system involvement	6 (11.3)	7 (9.3)	0.134	0.714
Skin and mucosal involvement	37 (69.8)	53 (70.7)	0.011	0.097
Neuropsychiatric involvement	6 (11.3)	3 (4)	2.546	0.111

### Laboratory test results in the noninfectious fever group and the no-fever group

The laboratory test results in the noninfectious fever group and the no-fever group were compared. The differences in low hemoglobin (*P*=0.036), leukopenia (*P*=0.025), CRP (*P*=0.035), complement C3 (*P*=0.036), albumin (*P*=0.039), and anti-dsDNA antibody (*P*=0.043) were statistically significant between the two groups, while the differences in thrombocytopenia (*P*=0.917), ALT (*P*=0.247), serum creatinine (*P*=0.499), and anti-SM antibody (0.866) were not statistically significant (Table 3).

**Table 3:** Laboratory test results in the noninfectious fever group and the no-fever group

***Laboratory test***	***Fever group (n=53) %***	***No-fever group (n=75) %***	***X ^ 2 ^ Value***	***P Value***
Low hemoglobin	36 (67.9)	37 (49.3)	4.380	0.036
Thrombocytopenia	16 (30.2)	22 (29.3)	0.011	0.917
Leukopenia	37 (69.8)	33 (44)	5.055	0.025
CRP	26 (49.1)	23 (30.7)	4.445	0.035
ALT	12 (22.6)	11 (14.7)	1.340	0.247
Serum creatinine	5 (9.4)	10 (13.3)	0.456	0.499
Complement C3	38 (71.7)	40 (53.3)	4.400	0.036
Decreased albumin	31 (58.5)	30 (40.0)	4.256	0.039
Anti-dsDNA antibody	35 (66)	36 (48)	4.091	0.043
Anti-SM antibody	17 (32.1)	23 (30.7)	0.029	0.866

### Medications in the noninfectious fever group and the no-fever group

The following common drugs used in clinical treatment of SLE were used in this study: glucocorticoids, cyclophosphamide, hydroxychloroquine/chloroquine, azathioprine/methotrexate and tripterygium glycosides. As shown in Table 4, more patients in the no-fever group were given glucocorticoids (*P*=0.027) and cyclophosphamide (*P*=0.034), compared to the patients in the fever group. The differences in the percentage of patients receiving these two drugs were statistically significant between the two groups. The percentages of patients receiving hydroxychloroquine/chloroquine (*P*=0.662), azathioprine/methotrexate (*P*=0.776) and tripterygium glycosides (*P*=0.527) were comparable in both groups, and the differences were not statistically significant.

**Table 4:** Medications in the noninfectious fever group and the no-fever group

***Medication***	***Fever group (n=53) %***	***No-fever group (n=75) %***	***X ^ 2 ^ /T Value***	***P Value***
Glucocorticoids	22 (41.5)	46 (61.3)	4.901	0.027
Cyclophosphamide	5 (9.4)	18 (24)	4.470	0.034
Hydroxychloroquine/chloroquine	7 (13.2)	12 (16)	0.192	0.662
Azathioprine/methotrexate	5 (9.4)	6 (8)	0.081	0.776
Tripterygium glycosides	7 (13.2)	13 (17.3)	0.401	0.527

### Multivariate analysis

Multivariate logistic regression analysis was performed on the general clinical data, clinical manifestations, laboratory test results, and medications for the 128 patients with SLE. The results showed that enlarged liver, spleen and lymph nodes, digestive system involvement, low hemoglobin, leukopenia, CRP, decreased albumin, anti-dsDNA antibody, glucocorticoids, and cyclophosphamide were associated with noninfectious fever in SLE (Table 5). The findings suggested that the above-mentioned factors were independent risk factors for noninfectious fever in SLE.

**Table 5:** Multivariate analysis of risk factors for noninfectious fever in SLE

***Factor***	***B***	***S.E.***	***Wald***	***df***	***Sig.***	***Exp(B)***
Enlarged liver, spleen and lymph nodes	−1.988	0.964	4.252	1	0.039	0.137
Digestive system involvement	−1.992	0.938	4.505	1	0.034	0.136
Low hemoglobin	−1.596	0.684	5.442	1	0.020	0.203
Leukopenia	−1.255	0.622	4.067	1	0.044	0.285
CRP	−1.371	0.647	4.496	1	0.034	0.254
Decreased albumin	−1.363	0.625	4.753	1	0.029	0.256
Anti-dsDNA antibody	−2.464	0.749	10.493	1	0.001	0.088
Glucocorticoids	1.934	0.683	8.026	1	0.005	6.919
Cyclophosphamide	3.069	0.998	9.456	1	0.002	21.522

## Discussion

SLE is an autoimmune disease that has multi-system and multi-organ involvement. Fever is one of the common systemic symptoms (5). Large sample studies indicated that fever accounted for 1.6% of initial symptoms of SLE. In a study reported by Al-Saleh et al, nearly half of 151 patients with SLE had fever (6). In this study, fever represented one of the leading causes for patients with SLE to be admitted to hospital, and as high as 41% of admitted patients had noninfectious fever. It should be noted that in addition to SLE disease activity, other extra conditions such as infection, tumor, and other noninfectious inflammatory disorders can also be a cause of fever. For example, prevalence of infection in SLE was approximately 30–40%. Thus, infection in SLE was a leading cause of fever and hospitalization, potentially leading to death (7). Administration of immunosuppressive agents was believed to be a major risk factor for secondary infection in SLE (8). When a fever occurs in SLE, the underlying cause should be differentially diagnosed. However, the differential diagnosis of infectious fever and noninfectious fever is difficult, due to lack of evidence-based criteria, especially for those patients who have a history of previous treatment with immunosuppressive agents. Occasionally, a misdiagnosis occurs and improper medications could be given to the patients, eventually causing the patients’ condition worsened. In recent years, there have been many studies on infectious fever in SLE and its comparison with disease activity-associated fever. This study was focused on exploration of the clinical characteristics of noninfectious fever in SLE through multivariate analysis of multiple risk factors, aiming to provide guidance for clinical diagnosis and treatment.

In this study, the average age of patients in the noninfectious fever group was 32.5 years, which was significantly lower than 36.7 years in the no-fever group, suggesting age was one of the causes of noninfectious fever in patients with SLE. Younger patients tended to have noninfectious fever. The percentage of initial treatment in the no-fever group (61.3%) was higher than that in the noninfectious fever group (43.4%), implying a history of previous treatment was a risk factor of noninfectious fever. It was reported that the SLEDAI score was associated with fever in SLE (9). This was consistent with the finding in this study, which showed the SLEDAI score was higher in the noninfectious fever group. The finding implied that higher disease activity often led to occurrence of fever.

Although SLE was more common in females, this study suggested that there was no gender difference in the prevalence of fever among patients with SLE. Except gender, family history and number of hospitalizations were not associated with fever either. In terms of clinical manifestations, the proportion of patients with enlarged liver, spleen and lymph nodes in the fever group (20.8%) was significantly higher than that in the no-fever group (8.0%), and the difference was statistically significant, indicating that enlarged liver, spleen and lymph nodes was associated with fever. The results also showed that the differences in proportion of patients with photosensitivity, arthritis, serositis, digestive system involvement, skin and mucosal involvement, and neuropsychiatric involvement were not statistically significant between the two groups. White blood cell count, as an indicator, is often used to differentiate an active fever. Infectious fever is usually indicated by an elevated white blood cell count, while non-infectious fever is indicated by a decreased white blood cell count. In this study, the proportion of patients with a decreased white blood cell count (leukopenia) was 44% in the no-fever group, while the proportion was 69.8% in the fever group. The difference was statistically significant.

CRP is another important indicator of the inflammatory response in the body. In this study, CRP in the fever group was significantly higher than that in the no-fever group, suggesting the inflammatory response was more pronounced in the fever group. The proportions of patients with low hemoglobin and with decreased albumin were both higher in the fever group than those in the no-fever group, suggesting there was a higher disease burden in the fever group. Therefore, patients with active fever should receive enhanced nutritional support. Possible association of liver and renal function with fever was examined by measuring serum levels of creatinine and ALT.

The results showed that proportion of patients with abnormal serum creatinine level in the no-fever group was higher than that in the fever group, suggesting that renal dysfunction was not associated with active fever. Although proportion of patients with abnormal ALT level in the fever group was higher than that in the no-fever group, abnormal liver function was not associated with active fever because the difference was not statistically significant between the two groups. In addition, patients in the fever group tended to have abnormal immune parameters, such as decreased complement C3 and positive anti-dsDNA antibody test.

At present, it is believed that active fever in SLE is due to activation of the monocyte-macrophage system in the body, which releases a large number of leukocyte pyrogens and produces prostaglandin E2, eventually causing dysfunction of the body temperature regulation center. Glucocorticoids suppress many functions of the activated monocyte-macrophage system. In view of concerns regarding adverse effects of various hormone drugs, it was recommended that hormone drugs should be used with caution in SLE (10). However, the choice drug for treating noninfectious fever in SLE is still prednisone, which is generally given to patients during the acute phase of SLE fever. The drug is usually administered in the early morning at a single dose of 1mg per kg per day, which is advisable for reducing the negative effects on the body. The dose can be reduced gradually when the fever subsides. A general guideline is that 5 mg of prednisone is reduced from the daily dose in the beginning until the dose drops to 30 mg, from when the daily decrease should be less and in the meantime close attention should be paid to the disease activity. In most cases the fever in SLE will subside when given a dose of ≤100 mg of prednisone per day. In rare cases the fever continues even a dose of >100 mg of prednisone is given. The numbers of monocytes and macrophages are larger in patients with SLE combined with multi-system infection than those in patients with active SLE. The corresponding cell activities are also stronger in patients with infection. Therefore, small to medium doses of glucocorticoids usually do not help in bringing the body temperature down in patients with infection. In this study, the proportion of patients with treatment history was higher in the fever group. However, smaller proportion of patients with treatment history received glucocorticoids and cyclophosphamide in the fever group. This finding implied that different prior medications could have inconsistent impact on the occurrence of fever.

SLE patients who received a maintenance dose or low to moderate dose of glucocorticoids were less likely to have active fever, whereas a high dose of hormone therapy may increase the risk of infectious fever (11). In another report, glucocorticoids and cytotoxic drugs were found to be major risk factors for infection in SLE (12). Therefore, in order to prevent disease recurrence and in the meantime avoid excessive drug use leading to infection, appropriate medications should be chosen for patients with SLE.

In this study, multivariate logistic regression analysis showed that factors, such as enlarged liver, spleen and lymph nodes, digestive system involvement, low hemoglobin, leukopenia, CRP, decreased albumin, anti-dsDNA antibody, glucocorticoids, and cyclophosphamide, were independent risk factors for noninfectious fever in SLE. These findings can further be verified in our future studies by using larger sample size and wider geographical distribution of patients. Statistical analysis of more patients from different geographical locations will provide more reliable results that can be used to guide clinical treatment. As found in this study, many risk factors can contribute to noninfectious fever in SLE. Thus, personalized treatment plans should be established using the results of evidence-based medical research and the specific conditions of patients. The goals are that patients can be differentially diagnosed early and receive timely treatment.

## Conclusion

The mechanism of fever in SLE is complex. The underlying causes can be mainly classified into two types: infection-induced fever and SLE disease activity-induced fever. Thus, the specific causes inducing fever in SLE should be actively looked into for individual patients. The use of hormone drugs should be careful and appropriate. For suspected patients with infectious fever, early differential diagnosis is expected to be done by examination of serum etiological antibodies and cultivation of pathogenic bacteria. In this study, the clinical manifestations of noninfectious fever in SLE, the patients’ general clinical data, laboratory test results and medications were explored using univariate analysis and multivariate logistic regression analysis. The goals of this study were to provide references for differential diagnosis, prevention and early intervention of fever in SLE and improve patient prognosis.

## Ethical considerations

Ethical issues (Including plagiarism, informed consent, misconduct, data fabrication and/or falsification, double publication and/or submission, redundancy, etc.) have been completely observed by the authors.
